# Automatic identification of suicide notes with a transformer-based deep learning model

**DOI:** 10.1016/j.invent.2021.100422

**Published:** 2021-06-24

**Authors:** Tianlin Zhang, Annika M. Schoene, Sophia Ananiadou

**Affiliations:** a Department of Computer Science, The University of Manchester, National Centre for Text Mining, Manchester, UK; b The Alan Turing Institute, London, UK

**Keywords:** Suicide notes, Social media, Deep learning, Natural language processing, Transformer-based model

## Abstract

Suicide is one of the leading causes of death worldwide. At the same time, the widespread use of social media has led to an increase in people posting their suicide notes online. Therefore, designing a learning model that can aid the detection of suicide notes online is of great importance. However, current methods cannot capture both local and global semantic features. In this paper, we propose a transformer-based model named TransformerRNN, which can effectively extract contextual and long-term dependency information by using a transformer encoder and a Bi-directional Long Short-Term Memory (BiLSTM) structure. We evaluate our model with baseline approaches on a dataset collected from online sources (including 659 suicide notes, 431 last statements, and 2000 neutral posts). Our proposed TransformerRNN achieves 95.0%, 94.9% and 94.9% performance in P, R and F1-score metrics respectively and therefore outperforms comparable machine learning and state-of-the-art deep learning models. The proposed model is effective for classifying suicide notes, which in turn, may help to develop suicide prevention technologies for social media.

## Highlights

• Early suicide note identification may help to develop suicide prevention technologies for social media.
• TranformerRNN was proposed, using the transformer encoder and BiLSTM.
• TranformerRNN can extract contextual information and latent features to identify suicide notes.
• Our TranformerRNN method outperforms comparable machine learning and state-of-the-art deep learning models.

## 1 Introduction

According to the World Health Organization (WHO), the total number of people dying from suicide is nearly 800,000 a year, and a recent study predicts the number is continually rising (Dhingra et al., 2015). Furthermore, suicide has become one of the leading causes of death (World Health Organization, 2014), which makes it a public health concern worldwide. Recently, social media platforms like Twitter and Facebook have become increasingly popular where people between 16 and 34 years old are more active (Chaffey, 2016). There also has been a growing trend that young people who potentially have suicide ideation leave their suicide notes on social media platforms (Desmet and Hoste, 2013; Ji et al., 2020; Luxton et al., 2012). Therefore, the automatic identification of suicide notes can play an important role in understanding people's mental health status and help to prevent suicidal behavior.

Previous works in identifying suicide notes used hand-crafted features and feature selection, including sentiment and linguistic features. For example, Jones et al. (Jones and Bennell, 2007) designed a classification model based on statistical prediction rules like average sentence length and other structural features. Pestian et al. (Pestian et al., 2010; Pestian et al., 2012) focused on emotion features and latent semantic features to identify suicide notes. In addition, some conventional machine learning algorithms such as Logistic Mode Tree (LMT) and Naive Bayes model are also used (Schoene and Dethlefs, 2016). Although these approaches have achieved some success, they rely heavily on feature engineering and costly expert knowledge from professionals such as forensic linguists and psychiatrists.

Deep learning allows models to automatically learn representations from data (LeCun et al., 2015) and has recently brought about a number of breakthroughs in natural language processing (Young et al., 2018), computer vision (Szegedy and Toshev, 2013) and speech recognition (Nassif et al., 2019). Moreover, some promising methods based on deep learning have been introduced to some mental health applications (e.g., depression detection (Acharya et al., 2018; Lam et al., 2019)) and achieved competitive performance. Sentiment analysis is concerned with detecting emotion and sentiment in textual data and is key for many Artificial Intelligence applications (Cambria, 2016). Early work related to sentiment analysis mainly focused on the linguistic feature selection using machine learning methods (Lin and Luo, 2020) (e.g., Support Vector Machine (SVM), Latent Dirichlet Allocation (LDA)) to improve the performance. More recently, deep learning approaches have become increasingly popular for a variety of sentiment analysis tasks. There are classic multiple neural network architectures (Zhang et al., 2018), including Convolutional Neural Networks (CNN), LSTM, LSTM with attention to extract subjective information. Cambria et al. (Cambria et al., 2020) built SenticNet6, a commonsense knowledge base, by using an ensemble of symbolic and sub-symbolic AI tools for sentiment analysis. Basiri et al. (Basiri et al., 2021) proposed an attention-based CNN-BiLSTM learning model to consider temporal information of texts. Li et al. (Li et al., 2020) designed a lexicon integrated two-channel CNN-BiLSTM model to improve performance. In addition, stacked ensemble learning (Akhtar et al., 2020) and multi-task learning (Majumder et al., 2019) are also used for sentiment analysis.

Similar to sentiment classification (Tang et al., 2015), deep learning is also a useful technique for identifying suicide notes, e.g., dilated LSTM with attention (DLSTMAttention) (Schoene et al., 2019). However, these methods cannot capture both local and global semantic features.

In this study, we propose a transformer-based deep learning model named TransformerRNN, which can extract contextual information and latent features to identify suicide notes by using the transformer encoder and BiLSTM. We evaluate the TransformerRNN using conventional machine learning methods and deep learning-based models on the same dataset. The results show that our model is better than baseline approaches on the suicide note identification task.

## 2 Dataset

### 2.1 Dataset collection

Identifying suicide notes is a subtask of text classification within the mental health domain. Besides suicide notes, we added last statements that were written by prison and a number of posts containing no obvious references to suicidal behavior. Therefore, in our experiments, the dataset covers suicide notes, last statements, and neutral posts, which is a 3-class classification task.

#### 2.1.1 Suicide notes

Some data was collected from existing corpora (Schoene et al., 2019), where it is known that the note writer has died by suicide. Due to the limited dataset size, we further extended our dataset with data collected from Kaggle.^1^

However, we do not know if a user who posted suicidal thoughts online has died by suicide. We used the Linguistic Inquiry and Word Count software (LIWC 2015) (Pennebaker et al., 2015) to compare the differences between the two datasets. LIWC (Pennebaker et al., 2015) has been developed to extract linguistic and psychological information via statical analysis based on word counts. We then use Cohen's d effect size (Cohen, 1992) for each feature between each dataset to calculate the statistical significance of each feature. We find that there are only a small number of features that have a medium effect size (the result of Cohen's d greater than 0.5), such as the emotions of a person, the usages of informal language and the second person pronoun, whereas all other linguistic features are similar. Therefore, we merge the two datasets from different sources creating a new dataset of 659 samples.

#### 2.1.2 Last statements

This data has been made available by the Department of Criminal Justices (Schoene et al., 2019), containing 431 records written prior to the death by prisoners who received death penalty between 1982 and 2017 in Texas totally.

#### 2.1.3 Neutral posts

The neutral posts dataset was collected from ten subreddits (e.g., r/fitness, r/parenting, r/teaching, r/relationships, etc.)^2^ where the posts did not contain obvious suicidal content. There is a total of 2000 samples in this corpus.

**Fig. 1:**
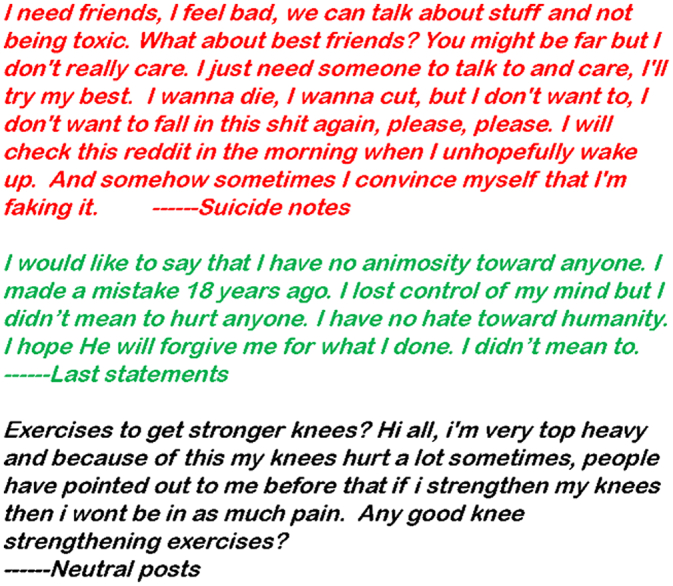
Examples of our dataset.

The data was collected from the public domain and we did not discriminate between gender or any other distinguished factors. To protect the authors' identity and preserve their privacy, we also removed personal information. Moreover, all data were also checked manually to ensure the accuracy of the label. Fig. 1 shows some examples of our dataset.

### 2.2 Dataset analysis

To better understand the linguistic clues and language usage of people who leave suicide notes behind, we analyzed our dataset in words, topics and other linguistic features.

Table 1 shows a quantitative comparison of our three corpora in terms of the number of notes and posts, the average number of words in each note and the average number of words in each sentence. It can be seen that the average note length of suicide notes is greater than others. Research by (Gregory, 1999) has shown that this could be due to people conveying their feelings as much as possible before they commit suicide. At the same time, the average number of words in a sentence of last statements is the lowest, which could be because people break their communication down into shorter units during stressful situations (Osgood and Walker, 1959), such as being a prison inmate on death row.

**Table 1:** Quantitative comparison of corpora.

Corpora	Suicide notes	Last statements	Neutral posts
No. of notes	659	431	2000
Av. no. of words in note	143.30	110.97	130.90
Av. no. of words in sentence	15.09	10.53	16.25

**Fig. 2:**
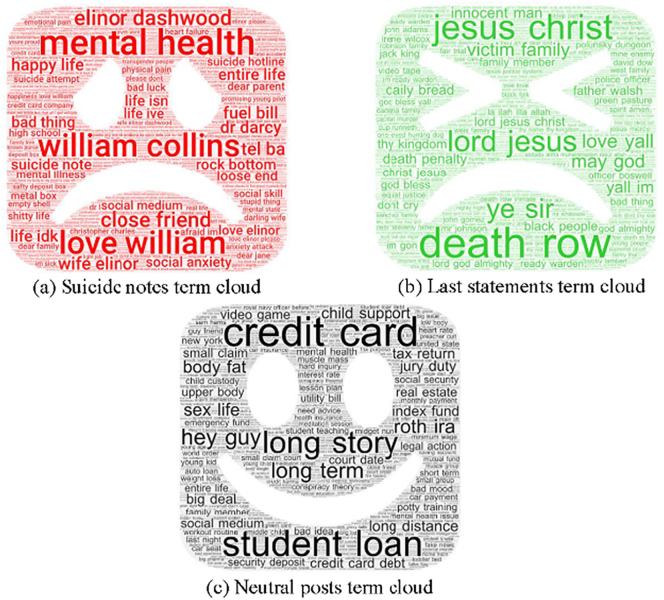
Term cloud visualization of our dataset, the term clouds were generated using the Termine system (Frantzi et al., 2000). http://www.nactem.ac.uk/software/termine/.

In addition, term clouds were used to compare the usage of high-frequency terms visually in different texts. The suicide notes frequently use some terms such as “mental health”, the mention of people (wife, William, friend etc.) and “life” as shown in Fig. 2(a), indicating that the writers have suicidal tendencies. Fig. 2(b) shows that last statement writers are showing their repentance by using “god”, “jesus christ” and “death row”. For example, someone wrote, “In the name of Jesus, I am sorry for the pain I caused you all.” For neutral Reddit posts, the dominant terms are mainly about everyday life like “student”, “credit card”, “story” and “guy”.

In order to show the different linguistic and psychological features in our datasets, we used the LIWC to analyze each type of note and post.

We also calculated effect sizes using Cohen's d (Cohen, 1992) between pairwise corpora to find linguistic features that are statistically significant (at least two results of Cohen's d greater than 0.5, because 0.2 indicates a small effect, 0.5 indicates a medium effect, and 0.8 indicates a large effect). As shown in Table 2, the listed items include dimension analysis, function and content word, affect analysis, social process, and personal concerns.

(i) The clout and tone for suicide notes are lowest, and last statements are highest overall. Clout refers to a person's social confidence or status in text (Pennebaker et al., 2014). Therefore, the results indicate that people who wrote suicide notes have a lower socio-economic status (Cohan et al., 2018). Tone stands for the emotional tone, where higher scores indicate greater emotional positivity (Cohn et al., 2004). The analysis of tone has also been verified in terms of affect analysis in Table 2, demonstrating that suicide notes express negative emotions (e.g., sadness, anxiety) and last statements often use resignation words (Schoene and Dethlefs, 2016).
(ii) The usage of function words and content words reflects how people communicate and what they say (Tausczik and Pennebaker, 2010). It has been observed that suicide notes and last statements use more personal pronouns because their authors prefer to focus on themselves (Just et al., 2017). We also compared the average number of adjectives and adverbs. The higher amount of these two parts of speech is observed in suicide notes, which means it is more likely that people tend to use more amplifying language (Baker and Baker, 2003), whereas the number of adjectives and adverbs in last statements is lower because prisoners have limited time to express their feelings (Hemming et al., 2020).
(iii) Social processes stand for the social relationships of writers, where we observe that in suicide notes writers tend to write less about social issues and family, while we observe the opposite in the results of last statements. The reason might be related to the low frequency in interpersonal relationships (Kelly and Foley, 2018).
(iv) Personal concerns highlight the common topics covered in notes. Unsurprisingly, most neutral posts refer to words related to work, and the topic of death is commonly referenced in suicide notes and last statements. Moreover, words related to religion are most referenced in suicide notes, which is confirmed by previous studies (Foley and Kelly, 2018) (Just et al., 2017).

**Table 2:** Linguistic statistical information extracted by LIWC.

Corpora	Suicide notes	Last statements	Neutral posts
Dimension analysis			
Clout	26.42	67.78	45.65
Tone	33.48	75.46	42.32

Function and content words			
Personal pronouns	15.29	19.75	11.33
Adjectives	4.42	2.54	4.04
Adverbs	6.45	3.09	5.47

Affect analysis			
Positive emotion	3.74	8.61	2.63
Negative emotion	4.06	2.55	1.97

Social processes			
Social	8.61	17.56	9.96
Family	0.75	2.11	0.85

Personal concerns			
Work	1.01	0.40	2.98
Religions	0.29	2.64	0.46
Death	1.28	0.68	0.15

## 3 Method

**Fig. 3:**
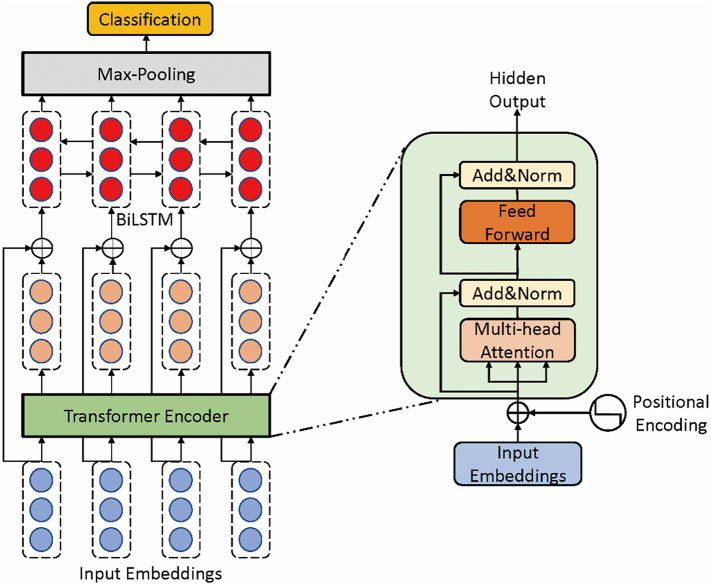
The overall architecture of TransformerRNN. The model contains five components: input embeddings, transformer encoder, BiLSTM, max-pooling layer and classification layer. The symbol ⊕ denotes vector concatenation. The internal architecture of transformer encoder is shown in light green block. More details about our model are provided in the main text.

In this section, we propose a Transformer-based Recurrent Neural Network (TransformerRNN) to identify suicide notes automatically. For this task, the input of the model is a note *N*, which is an input sequence of words *w*_1_, *w*_2_⋯*w*_*n*_. The output of the model is a predicted label *L* (suicide notes, last statements or neutral posts). The general architecture of TransformerRNN is shown in Fig. 3, which consists of five components: (1) input embeddings, (2) transformer encoder, (3) BiLSTM, (4) max-pooling layer and (5) a classification layer. In the following subsections, we will introduce each component of our model in detail.

### 3.1 Input embeddings

Word embeddings are the distributed representation of words, which are more suitable for natural language processing tasks and are used as input into neural networks (Bengio et al., 2003). In this paper, we use pretrained GloVe (Pennington et al., 2014) word representation for the word embeddings of inputs. Therefore, the input sequence is embedded into word vectors of *W* = {*w*_1_, *w*_1_⋯*w*_*n*_}, *W* ∈ *n* × *d*, where *n* is the length of note and *d* is the dimension of word embeddings.

### 3.2 Transformer encoder

Transformer encoders are a new type of sequence transduction model that can interactively calculate each word of the sequence to capture both local semantic and long-term dependency information without any convolutional or recursive structures (Vaswani et al., 2017). In this paper, we use the transformer encoder to model the input text.

The transformer encoder architecture contains the following components: multi-head self-attention layer, fully connected feed-forward network, layer normalization and positional encodings. The general architecture is shown as a light green block in Fig. 3.

Firstly, the positional encodings are added to the input embeddings to ensure that the model take advantage of the word-order or fixed sequential information, including relative and absolute positional information since there is no convolution or recurrence. In this work, we use sine and cosine functions of different frequencies proposed by Gehring et al. (Gehring et al., 2017) to get positional encodings.

The multi-head self-attention layer is the basic module of transformer encoder. The self-attention mechanism can be described as mapping a Query (*Q*) and a set of Key-Value (*K*—*V*) pairs to an output (Vaswani et al., 2017):

$$\mathrm{Attention}\left(Q,K,V\right)=\mathrm{softmax}\left(\frac{QK^{T}}{\sqrt{d_{k}}}\right)V$$

where *Q*, *K*, *V* and output are all matrices when a set of queries are computed simultaneously, and *d*_*k*_ is the dimension of queries and keys. Meanwhile, in order to allow the model to jointly gain information from different representation sub-spaces at different positions, multi-head self- attention is used.

$$\mathrm{MultiHead}\left(Q,K,V\right)=\mathrm{Concat}\left(\mathrm{hea}d_{1},\cdots \mathrm{hea}d_{h}\right)W^{O}$$

where *head*_*i*_ = *Attention*(*QW*_*i*_^*Q*^, *KW*_*i*_^*K*^, *VW*_*i*_^*V*^), *W*_*i*_^*Q*^ ∈ *ℝ*^*d*_*model*_×*d*_*k*_^, *W*_*i*_^*K*^ ∈ *ℝ*^*d*_*model*_×*d*_*k*_^, *W*_*i*_^*V*^ ∈ *ℝ*^*d*_*model*_×*d*_*v*_^ and *W*_*i*_^*O*^ ∈ *ℝ*^*nd*_*v*_×*d*_*model*_^, *h* is the number of heads, *d*_*k*_ = *d*_*v*_ = *d*_*model*_/*h*.

Next, the output of the multi-head self-attention layer is fed into a fully connected feed-forward network, which consists of two linear transformations with a Rectified Linear Unit (ReLU) (Li and Yuan, 2017) activation in between.

$$\mathrm{FFN}\left(x\right)=\text{ReLU}\left(W_{1}x+b_{1}\right)W_{2}+b_{2}$$

Additionally, the transformer encoder architecture contains a residual connection (He et al., 2016) and layer normalization (Ba et al., 2016) to accelerate the convergence speed.

### 3.3 BiLSTM layer

As shown in Fig. 3, we concatenate input embeddings and the hidden outputs of the transformer encoder so that the resulting representation contains both semantic information and contextual information. Then, we encode the transformer-based sequence via BiLSTM (Chen et al., 2017), which can not only capture long-term dependencies but also obtain context-aware information by modeling sequences from forward and backward hidden states. This BiLSTM contains a *forward LSTM* $\vec{\text{LSTM}}$ and a *backward LSTM* $\overset{\leftarrow }{\text{LSTM}}$, which learns sequence information from both directions.

$$\vec{h_{i}}=\vec{\text{LSTM}}\left(\vec{h_{i-1}},x_{i}\right),\overset{\leftarrow }{h_{i}}=\overset{\leftarrow }{\text{LSTM}}\left(\overset{\leftarrow }{h_{i+1}},x_{i}\right),h_{i}=\vec{h_{i}}⊕\overset{\leftarrow }{h_{i}}$$

where $\vec{h_{i}}\in {\mathbb{R}}^{h}$, and $\overset{\leftarrow }{h_{i}}\in {\mathbb{R}}^{h}$ (*h* is dimension of output) are hidden states of forward and backward LSTM at position *i*, respectively. *x*_*i*_ is the i-th input, ⊕ denotes concatenation. Finally, we obtain the encoding sequence as *H* = [*h*_1_, *h*_2_⋯*h*_*n*_].

### 3.4 Max-pooling layer and classification layer

After obtaining the output of the BiLSTM, we use it as direct input into the max-pooling layer. With the max-pooling operation, we can capture the most important latent semantic information throughout the note (Springenberg et al., 2014). Then, the last part of TransformerRNN is a classification layer (also called output layer), which is similar to traditional fully-connected layer. The prediction of probability distribution is calculated by using the softmax function:

$$P_{L}=\mathrm{softmax}\left(W_{3}H+b_{3}\right)$$

We train the model to minimize cross-entropy error:

$$\mathrm{Loss}=-\frac{1}{c}\sum_{i=1}^{c}t_{i}\log P_{L}$$

where *c* is the number of notes type and *t*_*i*_ ∈ {0, 1, 2} is the ground truth of label.

## 4 Results

We use precision (P), recall (R) and F1-score (F1) as complementary evaluation metrics to evaluate the model's classification performance on each class. We also use the weighted average metric method to show the overall performance. As shown in Table 3, the top, middle, and bottom parts are the machine learning-based baselines, the deep learning-based models and our model's results, respectively. The J48 Decision Tree (J48), Naive Bayes, Bayes Net and LMT were developed by using WEKA toolkit (Hall et al., 2009). Additionally, we also chose to benchmark our model also against CNN (Kim, 2014), BiLSTM (Schoene et al., 2019), BiLSTMAttention (Schoene and Dethlefs, 2018) and DLSTMAttention (Schoene et al., 2019) on the same datasets.

**Table 3:** The performance evaluation of different models on test set.

Method	Suicide notes			Last statements			Neutral posts			Avg.		
	P (%)	R (%)	F1 (%)	P (%)	R (%)	F1 (%)	P (%)	R (%)	F1 (%)	P (%)	R (%)	F1 (%)
J48	67.5	64.4	65.9	64.5	73.1	68.5	86.1	84.7	85.4	79.3	79.1	79.2
Naive Bayes	66.7	69.0	67.8	53.3	83.6	65.1	95.4	82.3	88.4	83.7	80.0	81.8
Bayes Net	88.1	67.8	76.6	66.3	94.0	77.8	93.5	91.0	92.2	88.4	87.0	87.7
LMT	82.6	65.5	73.1	100	65.7	79.3	87.7	**99.7**	93.3	88.5	88.1	88.3
CNN	90.0	72.4	80.2	93.9	91.0	92.4	91.9	97.7	94.7	91.8	91.9	91.7
BiLSTM	42.9	83.9	56.8	40.0	3.0	5.6	93.6	87.0	90.2	75.9	74.0	74.9
BiLSTMAttention	87.2	78.2	82.4	**96.9**	**92.5**	**94.7**	94.2	98.0	96.1	93.3	93.4	93.3
DLSTMAttention	85.5	81.6	83.5	**96.9**	**92.5**	**94.7**	94.8	97.0	95.9	93.3	93.4	93.3
TransformerRNN	**87.5**	**88.5**	**88.0**	94.0	94.0	94.0	**97.4**	97.0	**97.2**	**95.0**	**94.9**	**94.9**

Values in bold are the maximum scores attained.

We split the data into training, validation, and testing subsets with a proportion of 70%, 15%, 15%. We tune all parameters on the validation data, and the best performance results are reported based on test data. For tuned hyper-parameters of the TransformerRNN, we set the vector size of word embedding at 200, the initial learning rate as 0.0005, the dropout rate as 0.5, the dimension of BiLSTM hidden state as 128, the number of attention heads as 4, and the mini-batch size as 64.

**Fig. 4:**
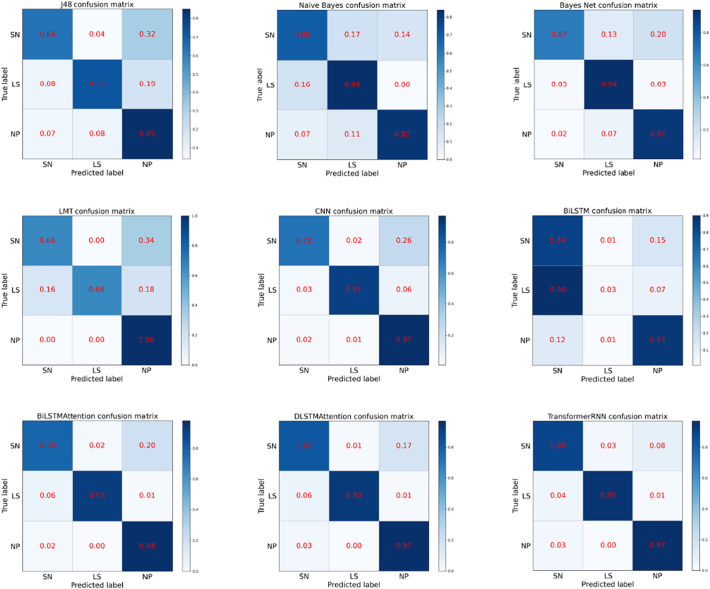
Confusion matrices for different models, SN stands for suicide notes, LS stands for last statements, NP stands for neutral posts.
(v) We also carried out ablation studies by removing components from the proposed TransformerRNN (Table 4). “No max-pooling” removed the max-pooling layer. “No BiLSTM” removed the BiLSTM part. “No concatenated embedding” removed word embeddings in the concatenated hid- den representation of transformer encoder. These results further prove the effectiveness of each component in our model.

The experimental results are summarized in Table 3, where we can observe that:

(i) In traditional machine learning models, LMT and Bayes Net classifiers gain relatively good performance, showing 88.3% and 87.7% in average F1-score. But the F1-scores of suicide notes are not high, with 73.1% and 76.6%, which shows that the conventional machine learning-based methods cannot capture the features of suicide notes effectively.
(ii) When we use deep learning methods, the results illustrate that neural network frameworks perform better at classifying suicide notes. For example, the CNN-based model achieves relatively good performance in F1-score. It is also observed that the BiLSTMAttention and DLSTMAttention outperform the traditional methods via attention mechanism, which makes them achieve 93.3% performance in average F1-score, and win 18.4% compared to a vanilla BiLSTM. This proves that neural network-based models with attention mechanism can make a significant contribution to suicide note classification by utilizing semantic representation.
(iii) Our proposed transformer-based model achieves the highest scores on suicide notes, neutral posts and overall performance. Compared with DLSTMAttention, TransformerRNN drops 0.7% in F1-score and 2.9% in P on last statements. However, our model has significant advantages in suicide notes classification which is more important for our task. Therefore, the results reveal our model can be useful to identify suicide notes and outperform existing state-of-the-art approaches.
(iv) In order to display classified results intuitively, we looked at the predicted labels in more details. Fig. 4 shows the normalized confusion matrices for different models over the test set. We observe that machine learning models often correctly predict neutral posts and misclassify suicide notes. For BiLSTM without attention mechanism, most last statements samples are misclassified into suicide notes.

**Table 4:** The performance evaluation of TransformerRNN and corresponding ablation studies.

Method	P (%)	R (%)	F1 (%)
TransformerRNN(ours)	95.0	94.9	94.9
No max-pooling	94.1	94.2	94.1
No BiLSTM	84.9	85.5	85.2
No concatenated embedding	93.3	93.2	93.2

## 5 Discussion

The purpose of this research is to design a model for suicide note classification, which could be useful in finding messages indicating potential suicidal behavior on social media platforms. Analyses from our dataset suggest that suicide notes have their own linguistic features. However, modeling with handcrafted identification rules is labor-intensive and costly. As seen in our experiments, our model outperforms all other baseline methods without any feature engineering. By encoding sentences with transformer encoder architecture, incorporating original word information, and capturing contextual information through BiLSTM, the TransformerRNN can better exploit the notes information from both syntactic and semantic aspects. Although the hybrid structure may increase some model complexity and the duration of training, users can use it to classify notes automatically once the model is well-trained.

There are also several potential limitations that are worth mentioning. First, the volume and sources of data are essential for training a stable and robust supervised learning-based model. In our dataset, the suicide notes collected are still insufficient (659 samples). Meanwhile, the Kaggle data is the text posted by users with a suicidal thought. Although these notes are similar to suicide notes in terms of linguistic features after LIWC analysis and also can help us understand people's mental status, it's not sure if users died by suicide. Thus, future studies should collect more precise data from different social media and groups of people. Additionally, semi-supervised and unsupervised approaches can be applied to suicide note identification. Second, unlike machine learning-based models, deep learning-based models have the advantages of automatic capturing semantic information and achieve remarkable performance, the drawback is that they are not directly interpretable. This is often not suitable for clinical decision-making process and needs to be taken into account when using such models. Despite these limitations, we believe that the application of deep learning in suicide note identification will have great development prospects.

## 6 Conclusions

We presented TransformerRNN, a transformer-based deep learning model, applied for suicide note identification. Our experiments demonstrated that our model outperforms conventional machine learning models and deep learning approaches on different datasets. The method proposed in this paper can be used as a means to suicidal risk identification from social media.

## Declaration of competing interest

The authors declare that they have no known competing financial interests or personal relationships that could have appeared to influence the work reported in this paper.

## Footnotes

1 https://www.kaggle.com/mohanedmashaly/suicide-notes/

2 https://www.reddit.com

## References

[bb0005] Acharya U.R., Oh S.L., Hagiwara Y. (2018). Automated EEG-based screening of depression using deep convolutional neural network[J]. Comput. Methods Prog. Biomed..

[bb0010] Akhtar M.S., Ekbal A., Cambria E. (2020). How intense are you? Predicting intensities of emotions and sentiments using stacked ensemble [application notes] [J]. IEEE Comput. Intell. Mag..

[bb0015] Ba J.L., Kiros J.R., Hinton G.E. (2016). Layer Normalization[J]. arXiv Preprint arXiv:1607.06450.

[bb0020] Baker M.C., Baker M.C. (2003). Lexical Categories: Verbs, Nouns and Adjectives[M].

[bb0025] Basiri M.E., Nemati S., Abdar M. (2021). ABCDM: an attention-based bidirectional CNN-RNN deep model for sentiment analysis[J]. Futur. Gener. Comput. Syst..

[bb0030] Bengio Y., Ducharme R., Vincent P. (2003). A neural probabilistic language model[J]. J. Mach. Learn. Res..

[bb0035] Cambria E. (2016). Affective computing and sentiment analysis[J]. IEEE Intell. Syst..

[bb0040] Cambria E., Li Y., Xing F.Z. (2020). SenticNet 6: Ensemble application of symbolic and subsymbolic AI for sentiment analysis[C]//. Proceedings of the 29th ACM International Conference on Information & Knowledge Management.

[bb0045] Chaffey D. (2016). Global Social Media Statistics Summary[J]. smartinsights. com.

[bb0050] Chen T., Xu R., He Y. (2017). Improving sentiment analysis via sentence type classification using BiLSTM-CRF and CNN[J]. Expert Syst. Appl..

[bb0055] Cohan A., Desmet B., Yates A. (2018). SMHD: A Large-scale Resource for Exploring Online Language Usage for Multiple Mental Health Conditions[J]. arXiv Preprint arXiv:1806.05258.

[bb0060] Cohen J. (1992). A power primer[J]. Psychol. Bull..

[bb0065] Cohn M.A., Mehl M.R., Pennebaker J.W. (2004). Linguistic markers of psychological change surrounding September 11, 2001[J]. Psychol. Sci..

[bb0070] Desmet B., Hoste V.R. (2013). Emotion detection in suicide notes[J]. Expert Syst. Appl..

[bb0075] Dhingra K., Boduszek D., O’Connor R.C. (2015). Differentiating suicide attempters from suicide ideators using the integrated motivational–volitional model of suicidal behaviour[J]. J. Affect. Disord..

[bb0080] Foley S.R., Kelly B.D. (2018). Forgiveness, spirituality and love: thematic analysis of last statements from Death Row, Texas (2002–17) [J]. QJM.

[bb0085] Frantzi K., Ananiadou S., Mima H. (2000). Automatic recognition of multi-word terms: the c-value/nc-value method[J]. Int. J. Digit. Libr..

[bb0090] Gehring J., Auli M., Grangier D. (2017). Convolutional sequence to sequence learning[C]//international conference on machine learning. PMLR.

[bb0095] Gregory A. (1999). The decision to die: the psychology of the suicide note[J]. Interviewing Deception.

[bb0100] Hall M., Frank E., Holmes G. (2009). The WEKA data mining software: an update[J]. ACM SIGKDD Explor. Newsl..

[bb0105] He K., Zhang X., Ren S. (2016). Deep Residual Learning for Image Recognition[C]//Proceedings of the IEEE Conference on Computer Vision and Pattern Recognition.

[bb0110] Hemming L., Pratt D., Shaw J. (2020). Prison staff’s views and understanding of the role of emotions in prisoner suicide and violence[J]. J. Forensic Psychiatry Psychol..

[bb0115] Ji S., Pan S., Li X. (2021). Suicidal ideation detection: a review of machine learning methods and applications[J]. IEEE Trans. Comput. Soc. Syst..

[bb0120] Jones N.J., Bennell C. (2007). The development and validation of statistical prediction rules for discriminating between genuine and simulated suicide notes[J]. Arch. Suicide Res..

[bb0125] Just M.A., Pan L., Cherkassky V.L. (2017). Machine learning of neural representations of suicide and emotion concepts identifies suicidal youth[J]. Nat. Hum. Behav..

[bb0130] Kelly B.D., Foley S.R. (2018). Analysis of last statements prior to execution: methods, themes and future directions[J]. QJM.

[bb0135] Kim Y. (2014). Convolutional neural networks for sentence classification[C]. Proceedings of the Conference on Empirical Methods in Natural Language Processing.

[bb0140] Lam G., Dongyan H., Lin W. (2019). Context-aware deep learning for multi-modal depression detection[C]//ICASSP 2019-2019. IEEE International Conference on Acoustics, Speech and Signal Processing (ICASSP).

[bb0145] LeCun Y., Bengio Y., Hinton G. (2015). Deep learning[J]. nature.

[bb0150] Li Y., Yuan Y. (2017). Convergence Analysis of Two-layer Neural Networks With Relu Activation[J]. arXiv Preprint arXiv:1705.09886.

[bb0155] Li W., Zhu L., Shi Y. (2020). User reviews: sentiment analysis using lexicon integrated two-channel CNN–LSTM family models[J]. Appl. Soft Comput..

[bb0160] Lin P., Luo X. (2020). A Survey of Sentiment Analysis Based on Machine Learning[C]//CCF International Conference on Natural Language Processing and Chinese Computing.

[bb0165] Luxton D.D., June J.D., Fairall J.M. (2012). Social media and suicide: a public health perspective[J]. Am. J. Public Health.

[bb0170] Majumder N., Poria S., Peng H. (2019). Sentiment and sarcasm classification with multitask learning[J]. IEEE Intell. Syst..

[bb0175] Nassif A.B., Shahin I., Attili I. (2019). Speech recognition using deep neural networks: a systematic review[J]. IEEE Access.

[bb0180] Osgood C.E., Walker E.G. (1959). Motivation and language behavior: a content analysis of suicide notes[J]. J. Abnorm. Soc. Psychol..

[bb0185] Pennebaker J.W., Chung C.K., Frazee J. (2014). When small words foretell academic success: the case of college admissions essays[J]. PLoS One.

[bb0190] Pennebaker J.W., Boyd R.L., Jordan K. (2015). The Development and Psychometric Properties of LIWC2015[R].

[bb0195] Pennington J., Socher R., Manning C.D. (2014). Glove: Global Vectors for Word Representation[C]//Proceedings of the 2014 Conference on Empirical Methods in Natural Language Processing (EMNLP).

[bb0200] Pestian J., Nasrallah H., Matykiewicz P. (2010). Suicide note classification using natural language processing: a content analysis[J]. Biomedical Informatics Insights.

[bb0205] Pestian J.P., Matykiewicz P., Linn-Gust M. (2012). What’s in a note: construction of a suicide note corpus[J]. Biomedical informatics insights.

[bb0210] Schoene A.M., Dethlefs N. (2016). Automatic identification of suicide notes from linguistic and sentiment features[C]//. Proceedings of the 10th SIGHUM Workshop on Language Technology for Cultural Heritage, Social Sciences, and Humanities.

[bb0215] Schoene A.M., Dethlefs N. (2018). Unsupervised Suicide Note Classification[C]// Workshop on Issues of Sentiment Discovery and Opinion Mining at Knowledge Discovery and Data Mining (KDD).

[bb0220] Schoene A.M., Lacey G., Turner A.P. (2019). Dilated lstm With Attention for Classification of Suicide Notes[C]//Proceedings of the Tenth International Workshop on Health Text Mining and Information Analysis (LOUHI 2019).

[bb0225] Springenberg J.T., Dosovitskiy A., Brox T. (2014). Striving for Simplicity: The All Convolutional net[J]. arXiv Preprint arXiv:1412.6806.

[bb0230] Szegedy C., Toshev A.D. (2013). Erhan. Deep neural networks for object detection [J]. Adv. Neural Inf. Proces. Syst..

[bb0235] Tang D., Qin B., Liu T. (2015). Document modeling with gated recurrent neural network for sentiment classification[C]//. Proceedings of the 2015 Conference on Empirical Methods in Natural Language Processing.

[bb0240] Tausczik Y.R., Pennebaker J.W. (2010). The psychological meaning of words: LIWC and computerized text analysis methods[J]. J. Lang. Soc. Psychol..

[bb0245] Vaswani A., Shazeer N., Parmar N. (2017). Attention is All You Need[J]. arXiv Preprint arXiv:1706.03762.

[bb0250] World Health Organization (2014). Preventing suicide: A global imperative[M].

[bb0255] Young T., Hazarika D., Poria S. (2018). Recent trends in deep learning based natural language processing[J]. IEEE Comput. Intell. Mag..

[bb0260] Zhang L., Wang S., Liu B. (2018). Deep learning for sentiment analysis: a survey[J]. Wiley Interdiscip. Rev. Data Min. Knowl. Discov..

